# Can parents reliably report burn scar quality on behalf of their child? A clinimetric observational study of child-proxy agreement on the pediatric patient scale of the POSAS

**DOI:** 10.1186/s41687-026-01140-y

**Published:** 2026-07-17

**Authors:** Frederique M. Kemme, Mieke Anthonissen, Annebeth Meij-de Vries, Pien Lelifeld, Thibau Demarbaix, Paul P. M. van Zuijlen, Anouk Pijpe

**Affiliations:** 1 Alliance of Dutch Burn Care (ADBC), Burn Center, Red Cross Hospital, Beverwijk, the Netherlands; 2 Amsterdam UMC, location Vrije Universiteit Amsterdam, Department of Plastic, Reconstructive and Hand Surgery, Amsterdam, the Netherlands; 3 Amsterdam Movement Sciences, Tissue Function and Regeneration, Amsterdam UMC, Amsterdam, the Netherlands; 4 OSCARE, Organisation for Burns, Scar After-Care and Research, Antwerp, Belgium; 5 University of Antwerp, Department of Rehabilitation Sciences and Physiotherapy, Research Group MOVANT, Antwerp, Belgium; 6 Department of Surgery, Red Cross Hospital, Beverwijk, the Netherlands; 7 Amsterdam UMC, location University of Amsterdam, Paediatric Surgical Center, Emma Children’s Hospital, Amsterdam, the Netherlands; 8 Department of Plastic, Reconstructive and Hand Surgery, Red Cross Hospital, Beverwijk, the Netherlands

**Keywords:** Proxy, Pediatric, Burn scars, POSAS, PROM, Proxy vs patient, Inter-rater reliability, Scar quality

## Abstract

**Background:**

The assessment of scar quality is an essential component of follow-up care and outcome evaluation in pediatric burn patients. The pediatric patient scale of the Patient and Observer Scar Assessment Scale (POSAS) is a recently developed patient-reported outcome measure (PROM) for this purpose. In pediatric care, proxy reports are commonly used when children are too young to self-report. However, the extent to which proxy reports reflect children’s own perception of scar quality is unclear. This study evaluated child-proxy agreement on the pediatric patient scale of the POSAS.

**Methodology:**

In this clinimetric observational study, children aged 8–17 years with burn scars and their proxies (parent or other caregivers) were recruited at the Burn Center in Beverwijk, The Netherlands, and aftercare center OSCARE in Antwerp, Belgium. Both the child and a proxy independently completed the pediatric patient scale of the POSAS, with proxies instructed to report from the child’s perspective *(proxy-patient perspective)*. Child and proxy item scores were described using medians and interquartile ranges (IQR) and compared using the Wilcoxon signed-rank test. Effect sizes were calculated to quantify the magnitude of differences. Agreement was examined at two levels: (1) presence or absence of complaints and (2) severity ratings. Subgroup analyses were performed for age category and proxy type.

**Results:**

A total of 81 child-proxy pairs were included between January and April 2025. Agreement on item level ranged from fair to substantial (kappa 0.309–0.692) and was moderate for most items. Overall agreement on the presence or absence of complaints was high (mean 78% concordance across items). Proxies reported worse scores than children for *color*,* bumpiness*,* thickness*,* tightness*, and *lines*, whereas children reported worse scores for *hair growth* (*p* < 0.05). Differences were generally small (≤1 response category). Agreement did not significantly differ across age groups or proxy type.

**Conclusions:**

In general, parents can report the presence or absence of their child’s scar complaints and can provide an indication of the severity of the complaint. Proxy reports can meaningfully contribute to scar assessment in pediatric burn care and research when self-report is not possible. The decision to use proxy reports should be guided by the intended purpose of measurement.

**Supplementary Information:**

The online version contains supplementary material available at https://doi.org/10.1186/s41687-026-01140-y.

## Background

Burn injuries are among the most common types of traumas in children and often result in scars, which can significantly impact physical function, psychosocial well-being, and overall quality of life [1, 2]. Consequently, the assessment of scar quality is an essential component of follow-up care and outcome evaluation in pediatric burn patients. It is typically assessed using clinician-reported outcome measures (CROMs), patient-reported outcome measures (PROMs), and measurement devices. Till recently, there was no appropriate pediatric-specific PROM to measure scar quality [3]. Therefore, the pediatric patient scale of the Patient and Observer Scar Assessment Scale (POSAS) is developed with input from pediatric burn patients and their caregivers (*Kemme et al.*,* revised version under review)*. The POSAS is a scar assessment scale that includes both patient- and observer perspectives and is suitable for assessing all types of scars [4, 5]. The recently developed *pediatric* patient scale includes twelve items covering visual, tactile, and sensory aspects of the scar that are relevant to children and is designed to complete independently from approximately 8 years old.

Nevertheless, one of the key challenges in pediatric burn patients is that the majority of the pediatric burn patients worldwide is very young (0–5 years old) [6], making self-report impossible. In these cases, proxy reports – typically provided by parents or other caregivers – are frequently relied upon in pediatric care. The use of proxies has been examined in a wide range of diseases and patient subgroups [7–11] and specifically in children [12–15]. A substantial body of literature shows imperfect agreement between child and parent assessments, a phenomenon known as cross-informant variance [16]. A systematic review of parent-child agreement across child health-related quality of life instruments found that agreement levels vary across domains and instruments, and that discrepancies are common [17]. Most studies have found that proxies rate health-related quality of life lower than patients [17–20].

Following the development of the pediatric patient scale of the POSAS and given the proportion of young burn patients, it is essential to determine how well proxies reflect the child’s perspective on burn scar quality. Therefore, this study aims to assess the agreement between child- and proxy reports on the pediatric patient scale of the POSAS. This is important to decide whether proxy reports should be used and if used, how scores should be interpreted [21].

## Methods

### Study design

This study is part of a larger research project aimed at validating the pediatric patient scale of the POSAS for children with burn scars. Ethical approval was obtained from the non-WMO Committee of the Medical Ethics Review Committee of Amsterdam University Medical Centers and the local Institutional Review Boards of the Red Cross Hospital and ZNA/OCMW Antwerp. The broader study investigates the measurement properties of the pediatric patient scale, and the reliability of the observer scale of the POSAS 3.0, which has been validated in adults but not yet in children. Within the overarching validation project, this clinimetric, observational study focuses on evaluating the reliability of proxy-reported scores by comparing proxy and child scar quality assessments of burn scars. Participating centers in this study are the Burn Center of the Red Cross Hospital in Beverwijk, The Netherlands, and Oscare – Center for Aftercare and Research in Antwerp, Belgium.

### Study population

Participants include Dutch speaking children aged approximately 8 to 17 years with a burn scar, who present with one of their parents or caregivers in the outpatient clinic and can independently complete the pediatric patient scale of the POSAS. Children from around 8 years of age can reliably self-report on their health [22–26]. Clinical experience guided inclusion of younger children only if they were deemed capable of completing the questionnaire meaningfully. Participants were excluded if they had insufficient proficiency in Dutch to complete the questionnaire, or if cognitive or mental impairments prevented them from completing the questionnaire. Following the COSMIN guidelines, a minimum of 50 participants were necessary to adequately assess the reliability of a patient-reported outcome measurement instruments [27]. We aimed to include 75 child-proxy pairs.

### Recruitment and data collection

Participants were recruited from the outpatient scar clinics of one of the participating centers. A pre-screening of scheduled outpatient visits was conducted beforehand to identify potential eligible participants. The treating physician introduced the study, after which the researcher provided detailed information about the study. Written informed consent was obtained from the proxy, and additionally from the child if aged 12 years or older (in the Netherlands). Data collection included proxy characteristics (relationship to the child; mother/father/other), patient characteristics (gender, current age), burn characteristics (post-burn day, etiology of the burn, % total body surface area (TBSA), and whether surgical wound closure was required in the acute phase. Subsequently, the child and proxy independently completed the pediatric patient scale at the same time. Proxies were explicitly instructed to complete the patient scale from their child’s perspective (*proxy-patient perspective*) [7]. The child and proxy were not allowed to discuss their responses or view each other’s answers during completion. After both questionnaires were finalized, they were permitted to review each other’s responses but were not allowed to make any changes.

### Pediatric patient scale of the POSAS

The scale consists of 12 items: *satisfaction*,* color*,* bumpiness*,* pain*,* thickness*,* tightness*,* itch*,* hardness*,* lines*,* numbness*,* hair growth*, and *burning sensation*. All items are scored on a 5-point emoticon response scale with 0 = ‘no, not at all’, 1 = ‘a little’, 2 = ‘so-so’, 3 = ‘quite a bit’, and 4 = ‘yes, very much’. (except the item ‘satisfaction’ which is reverse scored).

### Statistical analysis

All analyses were performed using IBM SPSS Statistics (version 30). Prior to analysis, all variables were checked for missing data and outliers. Descriptive statistics were used to describe data on proxy-, child- and burn characteristics.

#### Child and proxy item scores

For each item, median scores and interquartile ranges (IQRs) of children and proxies were calculated. Differences between child and proxy scores were assessed using Wilcoxon Signed Rank Test (*p* < 0.05). To quantify the magnitude of differences, effect sizes were calculated as r, defined as the standardized test statistic (Z) divided by the square root of the number of paired observations (N). Effect sizes were interpreted as small (≥ 0.1), medium (≥ 0.3), or large (≥ 0.5) [28].

#### Overall inter-rater agreement

Overall inter-rater agreement was quantified using quadratic weighted Cohen’s kappa (QWK) coefficients with 95% confidence intervals. QWK accounts for the ordinal response structure of the scale and the severity of discrepancies. Kappa values were interpreted according to established benchmarks: <0.20 (slight), 0.21–0.40 (fair), 0.41–0.60 (moderate), 0.61–0.80 (substantial), and > 0.80 (almost perfect agreement) [29].

#### Agreement on presence versus absence of a complaint

To evaluate agreement on the presence versus absence of a complaint, item scores were dichotomized into no complaint (score = 0) or a complaint (score > 0). Agreement was assessed using cross-tabulations of child and proxy responses. For each item, child–proxy pairs were classified into four categories: both reporting a complaint, both reporting no complaint, child reporting a complaint while the proxy did not, and proxy reporting a complaint while the child did not. Overall agreement on presence versus absence of complaints was calculated as the proportion of pairs in which child and proxy responses were concordant (both reporting a complaint or both reporting no complaint vs. one of them reporting a complaint while the other did not).

#### Direction of (dis)agreement

To compare severity scores between child and proxy, pairs were categorized into three groups: those with exact agreement (tie; child score = proxy score), proxy scoring worse than the child (proxy score > child score), and proxy scoring better than the child (proxy score < child score). For each item, the number and percentage of pairs per group were calculated.

#### Determinants of agreement

To explore whether discrepancies differed by age category (7–12, 13–15, ≥ 16 years) and proxy type (mother vs. father), we performed exploratory subgroup analyses. For continuous outcomes (child-proxy score differences per item), groups were compared using the Mann-Whitney U test. For dichotomous outcomes (agreement on presence or absence of a complaint), Fisher’s exact test was used. To explore whether certain pairs systematically demonstrated lower agreement on complaint presence or absence, an overall agreement score was calculated by summing binary agreement indicators across items. Higher scores reflected agreement on a greater number of items, whereas lower scores indicated more frequent disagreement on complaint presence or absence. To explore whether lower overall agreement clustered within specific subgroups, the total agreement score was examined in relation to child and burn characteristics.

## Results

### Participants

A total of 81 child-proxy pairs were included. Proxy-, child- and burn characteristics are displayed in Table 1. Most proxies were mothers (72%). Children’s gender was approximately evenly distributed (52% male, 48% female), and the median age was 12 years (IQR 10–15). Burns were most frequently caused by scalds (35%), followed by contact burns (19%) and firework (14%). Scar age ranged from one month to approximately 12 years, with a median of 9 months. Approximately half of the patients (54%) required surgery for wound closure.

**Table 1:** Characteristics of the study participants

	*N = 81*
**Children**	
**Gender**	
Female, n (%) Male, n (%)	39 (48%) 42 (52%)
**Current age, median (IQR, range)**	12 (10–15, 7–17)
Etiology of the burn, n (%)	
Scald Contact Firework Flame/fire Hot liquid/fat (no water) Chemical Other	28 (35%) 15 (19%) 11 (14%) 10 (12%) 9 (11%) 2 (2%) 6 (7%)
**Scar age (months), median (IQR, range)**	9 (3–83, 1-186)
**Surgery for wound closure, n (%)**	44 (54%)
**Proxies**	
**Relation to the child**	
Mother, n (%) Father, n (%) Other, n (%)	58 (72%) 20 (25%) 3 (4%)

### Child and proxy item scores

**Fig. 1:**
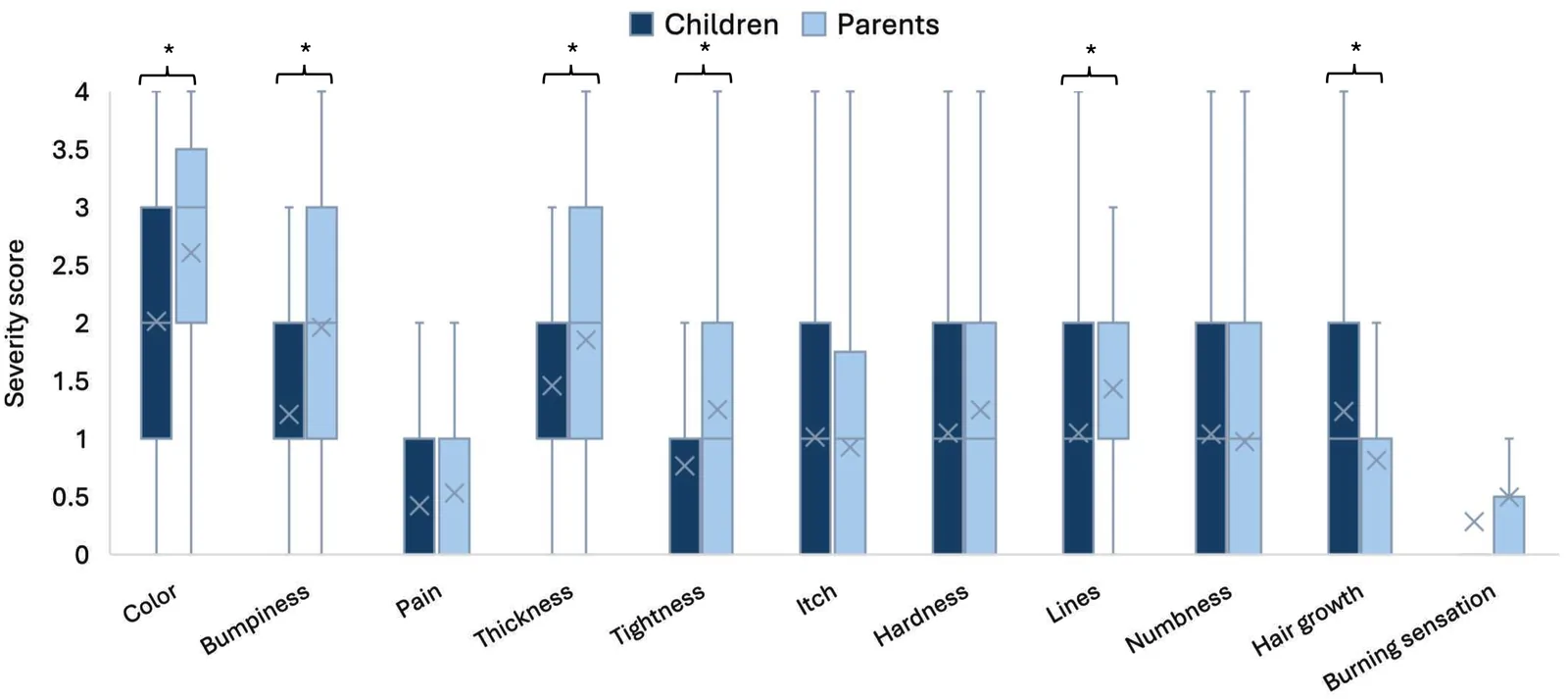
Median item scores (horizontal line) for children (dark blue) and proxies (light blue). *p < 0.05 (Related-Samples Wilcoxon Signed Rank Test). ‘X’ indicates the mean. Severity scores range from ‘no, not at all’ (0) to ‘yes, very much’ (4). Due to reverse scoring, the item ‘satisfaction’ is not shown

Median item scores for children (blue) and proxies (orange) are shown in Fig. 1. Proxies reported significantly worse scores than children for *color*,* bumpiness*,* thickness*, and *tightness* (*p* < 0.05). In contrast, children reported significantly worse scores than proxies for *hair growth* (*p* < 0.05). For *lines*, although the median was the same, proxy scores showed more higher values (median 1; IQR proxies 1–2 vs. children 0–2, *p* < 0.05). Moderate to large effect sizes were observed for *bumpiness* (*r* = 0.57), and moderate effects for *color* (*r* = 0.43), *thickness* (*r* = 0.30), *tightness* (*r* = 0.40), and *lines* (*r* = 0.33) (Supplementary Table 1). Small to moderate effects were found for *hair growth* (*r* = 0.27). Among pairs who both report a complaint, and without ‘ties’ (child score = proxy score), median absolute differences in severity scores were 1 for most items, 0.5 for *numbness*, and zero for *satisfaction*,* itch*, and *burning sensation*.

### Overall agreement

**Fig. 2:**
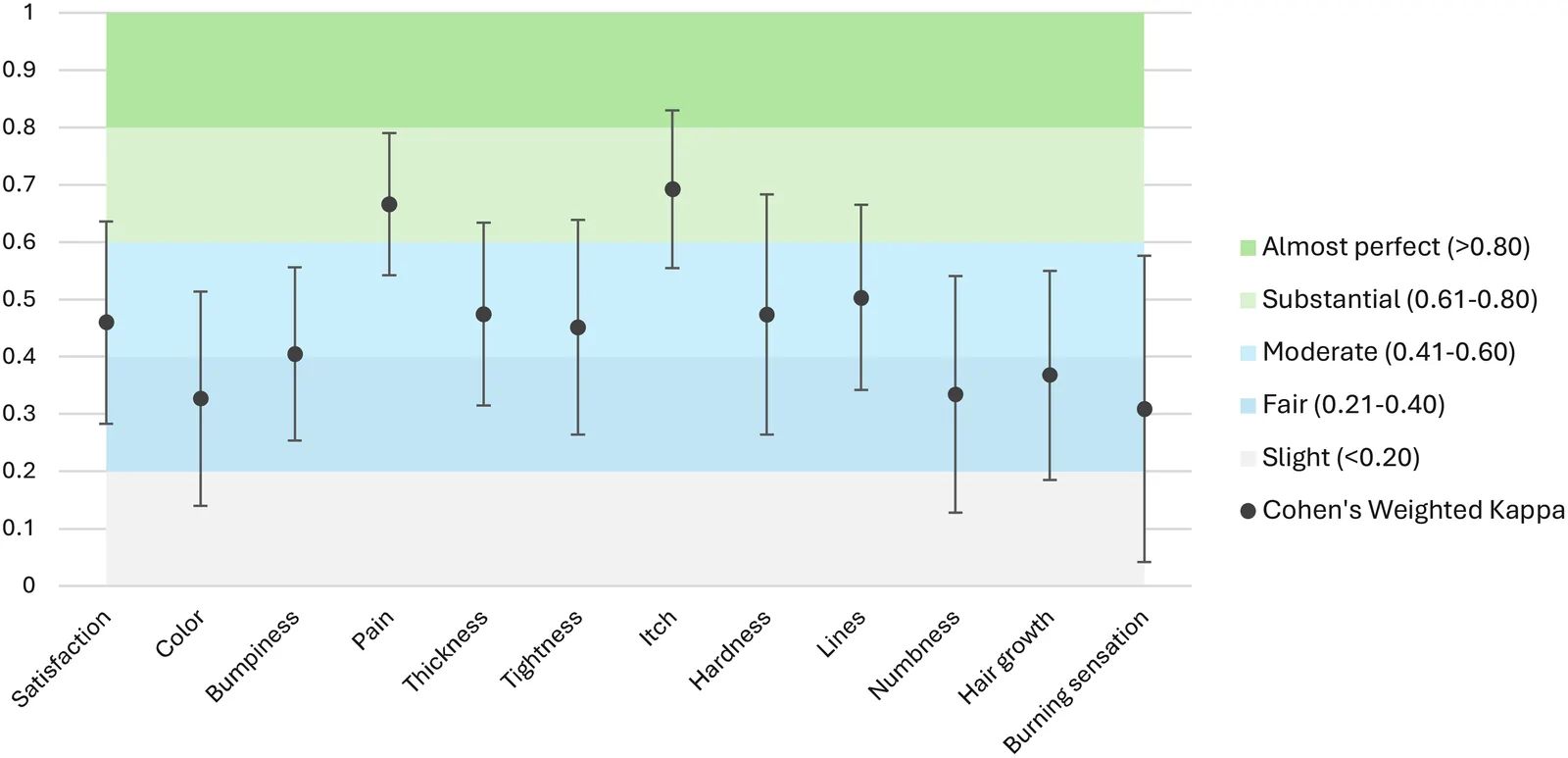
Quadratic weighted kappa values for each item^a^. ^a^Quadratic weighted kappa provides a chance-corrected measure of agreement that incorporates the severity of disagreement by weighting larger category differences more heavily than smaller ones

The proportion exact agreement between children and proxies varied across items and ranged from 32% for *color* to 77% for *burning sensation* (Table 2, column A). Quadratic weighted Cohen’s kappa values ranged from 0.309 to 0.692, reflecting fair to substantial agreement across items (Fig. 2). Agreement was highest (substantial) for *itch* (κ = 0.692) and *pain* (κ = 0.666). Moderate agreement was observed for *satisfaction*, *bumpiness*, *thickness*, *tightness*, *hardness*, and *lines* (κ = 0.40–0.50). Agreement was lowest (fair) for *color* (κ = 0.327), *numbness* (κ = 0.334), *hair growth* (κ = 0.368), and *burning sensation* (κ = 0.309).

### Agreement on the absence or presence of a complaint

**Fig. 3:**
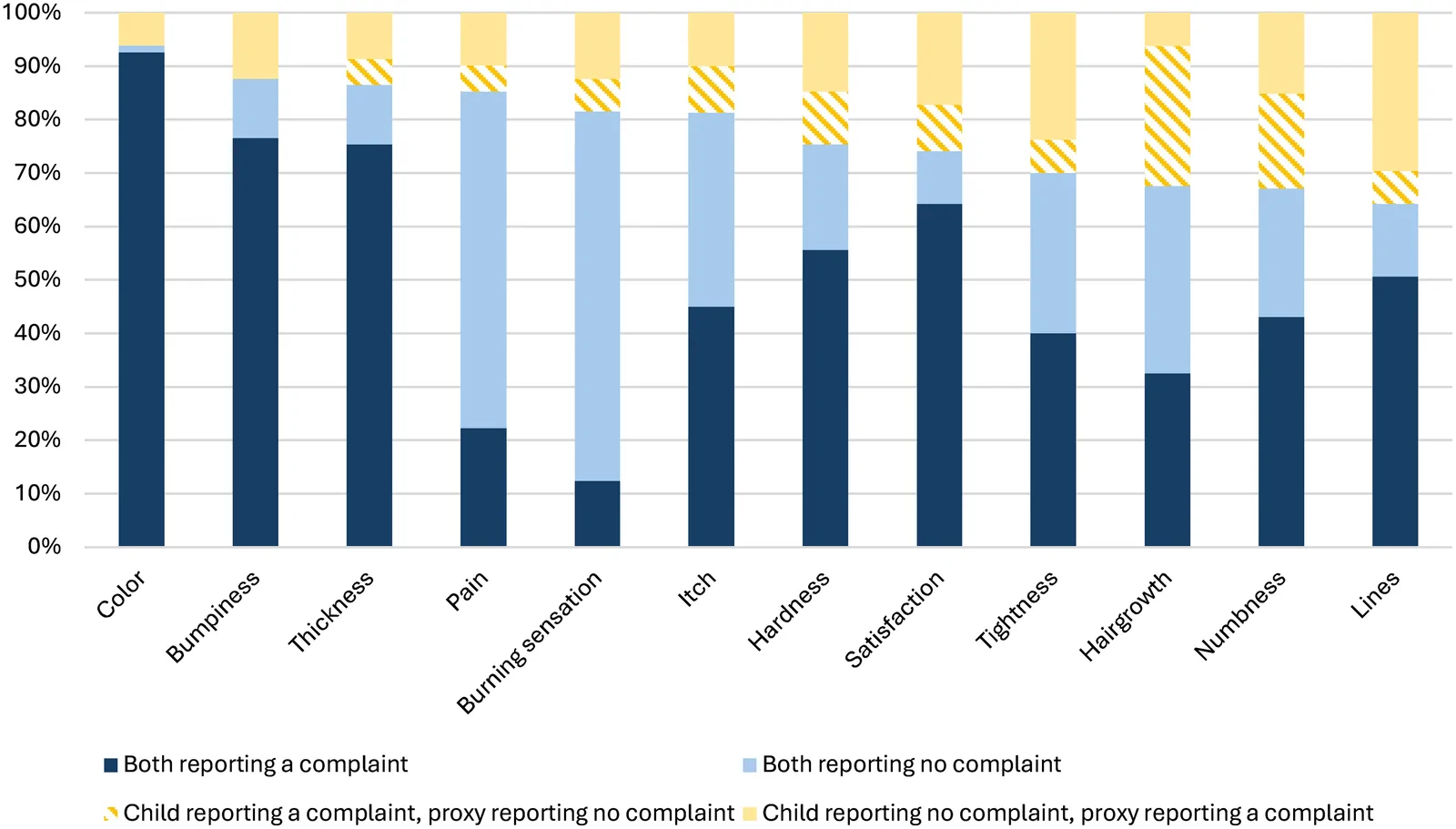
Proportion (%) agreement (blue) or disagreement (yellow) between children and proxies on the presence (dark blue and yellow striped) or absence (light blue and light yellow) of a complaint. Ordered from highest agreement (left) to lowest agreement (right)

Figure 3 visualizes the agreement between children and proxies on the absence or presence of a complaint per item. Overall, there was more agreement (blue) than disagreement (yellow), with a mean of 78% concordant responses compared with 22% discordant responses across items. Agreement was highest (> 80%) for *color*, *bumpiness*, and *thickness* (where most pairs reported a complaint), for *pain* and *burning sensation* (where most pairs reported no complaint), and for *itch* (where the distribution of complaints/no complaints was equal). In contrast, agreement was lowest for *hair growth*, *numbness*, and *lines*. Across most items, directional disagreement more frequently reflected proxies reporting a complaint while the child did not (light yellow). The reverse pattern (yellow) was observed for *hair growth* and *numbness*.

### Direction of (dis)agreement

To further explore the direction of (dis)agreement, pairs were divided into ties (A; child score = proxy score), proxies reporting worse scar quality than children (B), or proxies reporting better scar quality than children (C) (Table 2). Across nearly all items (except *itch*,* numbness*, and *hair growth)*, the proportion of pairs in which proxies reported worse scores than children (B) were higher than vice versa (C).

**Table 2:** Number and proportion of pairs with exact agreement (A; ties) or direction of disagreement per item (B, C)

	A		B		C	
	Child score = proxy score (ties)		Proxy score > child score		Proxy score < child score	
	N	*%*	N	*%*	N	*%*
Satisfaction	30	*37%*	30	*37%*	21	*26%*
Color	26	*32%*	40	*49%*	15	*19%*
Bumpiness	29	*36%*	45	*56%*	7	*9%*
Pain	57	*70%*	16	*20%*	8	*10%*
Thickness	30	*37%*	36	*44%*	15	*19%*
Tightness	33	*41%*	37	*46%*	10	*13%*
Itch	49	*61%*	15	*19%*	16	*20%*
Hardness	35	*43%*	30	*37%*	16	*20%*
Lines	29	*36%*	36	*44%*	16	*20%*
Numbness	36	*46%*	22	*28%*	21	*27%*
Hair growth	35	*44%*	14	*18%*	31	*39%*
Burning sensation	62	*77%*	14	*17%*	5	*6%*

Missing values: tightness n=1, itch n=1, numbness n=2, hair growth n=1

Percentages may not sum to 100% due to rounding

### Determinants of agreement

No significant differences in severity score difference and agreement on the presence or absence of a complaint were observed for age group and proxy type (data not shown).

## Discussion

Our findings provide insight into whether proxy reports can reliably reflect children’s perceptions of scar quality when self-report is not possible. Overall, agreement at item level was moderate for most items, with higher agreement for *pain* and *itch* and lower for *color*, *numbness*, *hair growth* and *burning sensation*. When examining agreement on the presence or absence of a complaint, concordance was generally high across items, with 78% agreement vs. 22% disagreement. This indicates that proxies can generally recognize whether their child experiences a complaint. Importantly, disagreement most frequently reflected proxies reporting a complaint that the child did not, although the reverse pattern also occurred. In terms of severity of a complaint, differences between child and proxy scores were generally small but significant for *color*,* bumpiness*,* thickness*, *tightness*, and *lines* (with proxies reporting worse scores than children), and *hair growth* (with children reporting worse scores than proxies). This pattern was also reflected in the direction of disagreement, as for most items, the largest proportion of pairs was observed in the category where proxies reported worse scar quality than children (except *itch*, *numbness*, and *hair growth*). However, discrepancies were bidirectional and cannot be interpreted as simple or systematic over- or underestimation by parents.

The interpretation of agreement statistics requires careful consideration. Previous research has demonstrated that even when “only” moderate patient-proxy correlations are observed at the item level, responses to individual items are identical or differ by only one response category (for questions with four or five response categories) in the vast majority (75–95%) of all patient-proxy comparisons. Large discrepancies tend to occur in only a minority of all comparisons made. Therefore, when interpreting agreement between patient and proxy ratings, it is recommended to include not only well-established measures of agreement but also straightforward proportions of agreement and adjacent-category differences [30, 31]. Importantly, discrepancies should not be interpreted as inaccuracies, but rather as reflecting complementary perspectives [18].

Research in pediatric health-related quality of life (HRQoL) provides relevant context [17, 32, 33]. Most of these studies consistently show that children’s perception of QoL differs from that of parents, with parents tending to rate children’s QoL lower than children themselves [34, 35], particularly in subjective domains such as emotional well-being, fatigue, and pain [34, 36–42]. In our study, proxies more frequently reported worse scar quality than children for most items, consistent with the broader literature. However, this pattern was not unidirectional, as children sometimes reported worse scores than their parents. This reinforces the notion that cross-informant variance reflects differences in perspective (although the use of a proxy-patient perspective) rather than consistent over- or underestimation. However, the observed discrepancies for *hair growth* – with children reporting worse scores than proxies – may partly reflect how children understand and interpret the item, potentially leading to variation in responses. Although this was not formally assessed in this study, the overarching validation study will further examine which items meaningfully contribute to the scale. Within the context of pediatric burn care, direct comparative studies are limited. Meyer et al. found overall good adolescent-proxy agreement on most domains of the Burn Outcomes Questionnaire (BOQ), though adolescents tended to report better outcomes regarding appearance and worse outcomes regarding itch compared to their proxies [20]. Similarly, Bamer et al. reported moderate agreement on PROMIS domains, with proxies typically rating greater symptom severity than children [18]. Our findings are broadly consistent with these studies: differences were small, and agreement varied by domain.

Agreement between child and proxy reports is influenced by a complex interplay of child-, proxy-, and methodological factors. Differences may arise because children and parents attend to different aspects of scar experience, including functional impact, visible appearance, and anticipated future consequences. Parents may compare the child’s condition to their pre-injury state, whereas children may be less likely to do so, particularly when they were very young at the time of the burn [30]. Emotional distress in parents, particularly following traumatic events like burn injuries, may potentially lead to an overestimation of symptoms [43]. On the other hand, increased family cohesion after a child’s burn injury, may partly explain higher levels of agreement [44]. Agreement may also be affected by methodological factors such as study design or psychometric properties of the instrument used [45]. For example, when the outcomes show little variation or are unevenly distributed, this can lower the observed agreement [46]. In addition, the extent to which proxies understand and adhere to reporting instructions is rarely assessed, yet may influence agreement [47]. In this context, proxy reporting can be categorized into two types: the *proxy-patient perspective* (used in this study), in which the proxy attempts to reflect how the child would rate their own condition, and the *proxy-proxy perspective*, which captures the proxy’s independent evaluation of the child’s health [7]. In our study, agreement did not significantly differ between age groups and proxy type. However, subgroup analyses for proxy type were limited by sample size particularly regarding the distribution between mothers and fathers. Previous research has suggested that fathers and mothers may differ in their perceptions of child health and that parental psychological factors, such as stress, may influence proxy ratings. For example, Pan et al. reported that higher parental stress was associated with less favorable parent-reported outcomes, and that father’s perceptions of appearance were related to their own post-traumatic stress levels [19].

This study has several strengths. It is the first study to examine agreement between child- and proxy-reported scar quality in pediatric burn patients. The pediatric patient scale of the POSAS used was specifically developed with and for pediatric burn patients. This makes it highly relevant and specific for the target population. Also, it had a relatively large sample size, and we performed extensive statistical analyses. However, several limitations should be acknowledged. First, evaluating child-proxy agreement inherently requires children who can self-report, which restricted our sample to those aged approximately eight years and older. While methodologically unavoidable, this means that agreement could not be assessed in younger children – the group for whom proxy reports are most often used. Consequently, this limits direct generalizability and creates some uncertainty about whether similar levels of agreement would be observed for younger children. Second, limited information was collected regarding proxy characteristics, limiting the exploration of potential determinants of discrepancies. Although not considered a limitation of this study, it should be acknowledged that although proxies were explicitly instructed to fill out the patient scale from the perspective of the child, it is likely that their own interpretations and concerns influenced responses regardless.

From a clinical perspective, a key question is what the intended purpose of proxy reporting is. If the aim is to obtain a substitute measure that can stand in for the child’s own report, high levels of agreement are crucial and differences between reporters must be carefully understood. However, if the goal is to obtain a richer and more comprehensive understanding of the child’s experience though multiple perspectives, then discrepancies are not necessarily problematic and may in fact be informative [17]. In routine burn care follow-up, the pediatric patient scale of the POSAS is primarily used for monitoring and evaluation, rather than for clinical decision making. Proxy reports therefore appear to provide a sufficiently reliable patient-perspective estimate. However, greater caution is warranted when the pediatric patient scale is used as a primary outcome measure in research. Based on our findings, proxy reports can meaningfully contribute to scar assessment in pediatric burn care when self-report is not possible. We recommend analyzing child self-reports and proxy reports separately, or combined analyses supplemented by sensitivity analyses on self-reports. In longitudinal studies, switching raters over time (e.g. proxy-report at baseline and child-report at follow-up) should be avoided. Depending on the purpose of a study, proxy-reported scores may be less suitable as the primary outcome. Additional approaches with objective outcome measurement instruments, such as CROMs (e.g., the POSAS observer scale), may be considered.

## Conclusion

Using the pediatric patient scale of the POSAS, proxy reports generally provide a reliable estimate of children’s perceived scar quality, particularly for identifying the presence or absence of complaints. Although exact agreement on severity ratings is less frequent, differences are typically small. While proxies more often rated scar quality as worse than children did, disagreement was bidirectional and can therefore not be interpreted as systematic bias. Proxy reports can meaningfully contribute to scar assessment in pediatric burn care when self-report is not possible. However, they should not be considered fully interchangeable with child self-report. Ultimately, the decision to use proxy reports should be guided by the intended purpose of the measurement.

## Supplementary Information

Below is the link to the electronic supplementary material.

Supplementary Material 1

## Data Availability

All data generated or analysed during this study are included in this published article. The datasets used and/or analysed during the current study are available from the corresponding author on reasonable request.
