# Supplementary material for: Can parents reliably report burn scar quality on behalf of their child? A clinimetric observational study of child-proxy agreement on the pediatric patient scale of the POSAS
Source: J Patient Rep Outcomes. 2026 Jul 17;10:188. doi: 10.1186/s41687-026-01140-y (PMC13658675; doi:10.1186/s41687-026-01140-y)
Supplement: Supplementary file 1 [file 41687_2026_1140_MOESM1_ESM.docx]

**Supplementary Table 1.** Median scores and differences between child and proxy item scores

| Item | Median proxy score^a^ | Median child score^a^ | p-value of the difference^b^ | Effect size *r^c^* |
| --- | --- | --- | --- | --- |
| Satisfaction^d^ | 1 | 1 | 0.274 | 0.12 |
| Color | 3 | 2 | **<0.001** | **0.43** |
| Bumpiness | 2 | 1 | **<0.001** | **0.57** |
| Pain | 0 | 0 | 0.162 | 0.16 |
| Thickness | 2 | 1 | **0.007** | **0.30** |
| Tightness | 1 | 0 | **<0.001** | **0.40** |
| Itch | 1 | 1 | 0.531 | 0.07 |
| Hardness | 1 | 1 | 0.116 | 0.17 |
| Lines | 1 | 1 | **0.003** | **0.33** |
| Numbness | 1 | 1 | 0.880 | 0.02 |
| Hair growth | 0 | 1 | **0.014** | **0.27** |
| Burning sensation | 0 | 0 | 0.052 | 0.22 |

^a^Among all participants

^b^Calculated by the Related-Samples Wilcoxon Signed Rank Test

^c^Classified as small (≥ 0.1), medium (≥ 0.3), or large (≥ 0.5) [23].
^d^The item ‘satisfaction’ is reverse scored (0 = ‘yes, very much’ 4 = ‘no, not at all’), for all subsequent items 0 indicates ‘no, not at all’ and higher scores indicate greater symptom severity.
